# Influence of functional task-oriented mental practice on the gait of transtibial amputees: a randomized, clinical trial

**DOI:** 10.1186/s12984-017-0238-x

**Published:** 2017-04-11

**Authors:** Rodrigo Gontijo Cunha, Paulo José Guimarães Da-Silva, Clarissa Cardoso dos Santos Couto Paz, Ana Carolina da Silva Ferreira, Carlos Julio Tierra-Criollo

**Affiliations:** 1 Graduate Program in Neuroscience—Federal University of Minas Gerais, Avenue Presidente Antônio Carlos, 6627 Belo Horizonte, Brazil; 2 Engineering School, Center for Research and Education in Biomedical Engineering—Pampulha, Belo Horizonte, MG 31270-901 Brazil; 3 Alberto Luiz Coimbra Institute for Graduate Studies and Research in Engineering, Biomedical Engineering Program, Federal University of Rio de Janeiro, Rio de Janeiro, Brazil; 4 Faculty of Ceilândia, University of Brasília, Brasília, Brazil; 5 Biomechanics Laboratory of Federal University of Minas Gerais, Federal University of Minas Gerais, Belo Horizonte, Brazil

**Keywords:** Mental practice, Motor imagery, Rehabilitation, Lower limb amputees, Gait

## Abstract

**Background:**

Mental practice (MP) through motor imagery is a cognitive training strategy used to improve locomotor skills during rehabilitation programs. Recent works have used MP tasks to investigate the neurophysiology of human gait; however, its effect on functional performance has not been evaluated. In the present study, the influence of gait-oriented MP tasks on the rehabilitation process of gait in transtibial amputees was investigated by assessing the vertical (V), anterior-posterior (AP), and medio-lateral (ML) ground reaction forces (GRFs) and the time duration of the support phase of the prosthetic limb.

**Methods:**

Unilateral transtibial amputees, who were capable of performing motor imagination tasks (MIQ-RS score ≥4), were randomly divided into two groups: Group A (*n* = 10), who performed functional gait-oriented MP combined with gait training, and Group B (*n* = 5), who performed non-motor task MP. The MP intervention was performed in the first-person perspective for 40 min, 3 times/week, for 4 weeks. The GRF outcome measures were recorded by a force platform to evaluate gait performance during 4 distinct stages: at baseline (BL), 1 month before the MP session; Pre-MP, 1–3 days before the MP session; Post-MP, 1–3 days after the MP session; and follow-up (FU), 1 month after MP session. The gait variables were compared inter- and intra-group by applying the Mann-Whitney and Friedman tests (alpha = 0.05).

**Results:**

All volunteers exhibited a homogenous gait pattern prior to MP intervention, with no gait improvement during the BL and Pre-MP stages. Only Group A showed significant improvements in gait performance after the intervention, with enhanced impact absorption, as indicated by decreased first V and AP peaks; propulsion capacity, indicated by increasing second V and AP peaks; and balance control of the prosthetic limb, indicated by decreasing ML peaks and increasing duration of support. This gait pattern persisted until the FU stage.

**Conclusions:**

MP combined with gait training allowed transtibial amputees to reestablish independent locomotion. Since the effects of MP were preserved after 1 month, the improvement is considered related to the specificity of the MP tasks. Therefore, MP may improve the clinical aspect of gait rehabilitation when included in a training program.

## Background

Human gait is characterized by synchronous, regular, and successive movements that are controlled by complex neural pathways. The cerebral cortex transmits the motor commands to the peripheral nervous system in order to coordinate the contractions of 57 muscles and the movements of 11 joints to generate a sequence of kinetic forces for locomotion [1–3]; this can be evaluated by the ground reaction force (GRF) through a force platform [1–7]. Weight acceptance, single limb support, and limb advancement are specific functional tasks related to the normal walking pattern. However, the motor pattern of these tasks is altered in unilateral transtibial amputees, changing the natural cadence [4, 5]. After limb amputation, changes in motor ability due to body alignment of the trunk and knee position and due to the anterior tilt of the socket lead to an alteration of the center of mass over the support base. Besides, lower limb amputees spend less time in the stance phase to minimize any discomfort, pain, or instability to support the body weight on the prosthetic side [4–6]. Moreover, the functional loss of the ankle modifies the mechanical properties for controlling weight acceptance and the forward rotation of the supporting limb [1, 2, 4, 5, 7], consequently reducing the mechanical power during the push-off phase.

Generally, physical therapy using prosthetic training programs is based on muscle strengthening activities and proprioception exercises, including balance and postural control tasks to improve gait function in amputees after surgery [3, 8–11]. Lower limb amputees transfer impact-absorption and proprioceptive information to the discharge point at the residual limb in contact with the prosthesis socket [4, 10, 12, 13]. Thus, one of the difficulties of gait rehabilitation in lower limb amputees is the lack of synchrony in proprioception, as appropriate information is not received, which makes it hard to adapt to the force acting at the residual limb during the support phase. Moreover, an asymmetrical gait pattern with greater oscillation of the center of mass towards the side of the remaining limb during stance leads to higher energy consumption [3–5, 9, 14, 15]. On the other hand, maintaining an equal step length is the most difficult element in gait training in transtibial amputees, since their biomechanical capabilities and constraints are asymmetric [5, 14, 15]. Therefore, the clinical aspect of gait rehabilitation and physical therapy procedures aims to minimize greater oscillation of the center of mass, energy expenditure, and/or pain, even if the gait pattern remains asymmetrical.

Various studies [16–21] have shown that the central nervous system exhibits significant plasticity after an injury, particularly after limb amputation [17, 19]. The absence of the input/output from the missing limb reduces the representation map of both the primary motor and somatosensory cortex areas. On the other hand, different rehabilitation techniques and approaches, such as passive and active exercises, observation or imitation actions, as well as functional task-oriented mental practice (MP), can affect such plasticity at distinct levels, including at the neuroanatomical and functional organization, physiological, cell, molecular, and behavioral levels [16–18, 20–23]. Thus, the neural correlates of motor planning during MP tasks and movement depend on the amount of motor-sensory experience to preserve limb representation.

MP is a cognitive strategy procedure based on extensively repeated motor imagery tasks, which can enhance the acquisition of motor ability and functional performance, without physical execution of movements [24–33]. This procedure activates specific encephalic areas related to the motor planning and preparation of the motion (thinking before execution) and is used to promote motor learning of daily living tasks [20, 26, 28, 31]. Thus, MP of locomotor skills can activate endogenous representations related to specific movements, including gait [27, 28, 30, 33]. Such approaches can be adopted via two perspectives: (1) third-person perspective, wherein the individual imagines another person walking (i.e., the individual imagines as if he/she were watching the movement being performed), or (2) first-person perspective, wherein the individual imagines him/herself performing the gait [24, 26, 27]. Additionally, MP performed in the first-person perspective can generate additional cognitive processes, enhance internal sensory activation without any motor output [25–27, 30], and enable kinesthetic representation of the gait phases [27, 33].

Although MP has been used as a motor imagery strategy to improve the performance of a motor task in sport or locomotor skills during neurological rehabilitation [25, 32, 34–36], including for functional gait movements [26, 27, 30, 31], this technique has not been widely used in the rehabilitation of transtibial amputees. When MP is used as a training tool during rehabilitation, the cognitive process established during the mental task can enhance both motor planning and motor execution [25, 28]. Moreover, MP combined with physical practice has been shown in several studies to be more efficient for generating specific motor skills in order to control movements used in activities of daily life [25, 28–31, 36]. Based on these previous findings, our study of MP intervention during rehabilitation of transtibial amputees is clinically relevant and justified, as this approach can be hypothesized to improve their motor performance in terms of controlling the movements of the prosthetic limb, especially during the initial contact and weight acceptance phases of gait. By assessing the first peak of the vertical GRF (V1, load response as a percentage of body weight) using a force platform, it is possible to evaluate the gait performance as a result of the manner in which the individual touches the ground to preserve the forward progression and maintain stability during prosthetic limb support [1, 2, 6, 37–39]. Moreover, the improvement of these functional pattern phases is important to generate forward force propulsion (V2 GRF peak), as well as limb advancement [1, 2, 6, 37, 39].

Accordingly, in the present study, we aimed to analyze the influence of gait-oriented MP in the rehabilitation process of transtibial amputees through the assessment of kinetic variables (vertical, anterior-posterior, and medio-lateral GRFs) and the time duration of the support phase of the prosthetic limb. Specifically, we proposed the following hypotheses: (1) gait-oriented MP intervention in transtibial amputees (the experimental group) will significantly improve the functional performance of gait and equalize the kinetic variables, leading to better control of the prosthetic limb movement; (2) gait-oriented MP intervention in transtibial amputees will significantly improve the duration of the support phase; and (3) the kinetic variables will not differ before and after the intervention in the control group (non-gait-oriented MP intervention).

## Methods

In this randomized clinical study, we assessed the effects of applying motor-task MP to improve the functional performance of gait in transtibial amputees. Based on previously established inclusion criteria, we enrolled unilateral transtibial amputees, aged between 18 and 60 years, with a time since amputation of 1–40 years, who were capable of performing motor imagination tasks, as evaluated by the Motor Imagery Questionnaire-Revised, Second Edition (MIQ-RS) [34]. In addition, none of the volunteers presented: A) a previous history of lower limb surgery; B) rheumatic, orthopedic, or neurological diseases with motor sequelae; C) vestibular and/or cerebellar disorders; D) serious hearing and/or visual impairment that had not been corrected; or E) heart disorders that might influence gait.

**Fig. 1:**
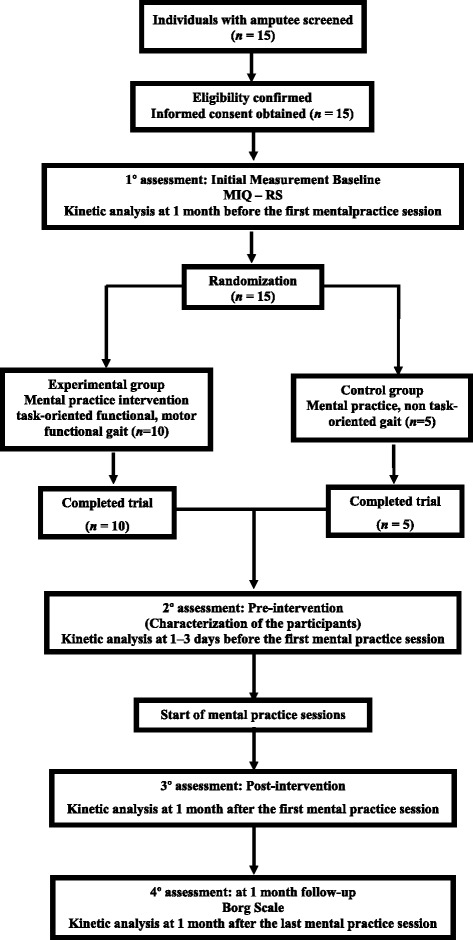
Flowchart of the study protocol. MIQ-RS, Motor Imagery Questionnaire-Revised

Figure 1 shows a flowchart of the study design and study protocol. The volunteers were recruited from clinical and hospital associations in Minas Gerais, Brazil. After screening and obtaining consent (ethical approval #0591.0.203.000-0) from the Research Ethical Committee of the Federal University of Minas Gerais, Brazil, demographic, anthropometric, and clinical data were collected from 16 male transtibial amputee volunteers.

All volunteers presented an MIQ-RS score of ≥4, which indicated their ability to perform visual motor and kinesthetic imagination tasks [34] and, hence, they were included in the study. The volunteers were randomly divided into two groups: Group A (*n* = 8) and Group B (*n* = 8). Group A performed a gait-oriented MP task, while Group B performed a non-motor MP task. The preliminary analysis, which was performed with 5 volunteers per group, indicated a benefit gain in gait function only for Group A (statistical power: 89%; effect size: 2.0; alpha = 5%). Since there is no known risk of using mental practice, it would not be scientifically ethical to expose the volunteers to a specific intervention that did not support the significant results seen in gait function. Therefore, based on this benefit, the remaining three volunteers from Group B were subsequently included in Group A. However, one volunteer did not complete the study. Thus, Group A comprised 10 volunteers (age range, 20–46 years; mean age, 33.2 ± 2.69 years), with an average time since amputation of 15.5 ± 2.1 years (range, 8–26 years), and presenting with an average visual score of 22.7 ± 1.76 and kinesthetic score of 22.3 ± 2.66. Group B remained with 5 volunteers (age range, 26–45 years; mean age, 35.4 ± 3.2 years), with average time since amputation of 24.4 ± 2.92 years (range, 18–34 years), and presenting with an average visual score of 22 ± 2.91 and kinesthetic score of 22.2 ± 2.58.

The experimental design used in this study was A_1_-B-A_2,_ which is common practice in rehabilitation studies, when the therapist is faced with stabilization of the patient and need to implement treatment strategies to increase the functional capacity of these individuals. Phase A_1_ did not include any intervention (MP or conventional physical therapy treatment), Phase B included MP intervention, and Phase A_2_ did not include any intervention (MP or conventional physical therapy treatment).

Each phase (A_1_, B, and A_2_) had a time duration of 4 weeks (1 month). The GRF outcome measures were recorded by evaluating the gait performance during 4 distinct stages: (*i*) baseline (BL), occurring during the beginning of phase A_1_, 1 month before the first MP session; (*ii*) Pre-MP, occurring at the final of phase A_1_, 1–3 days before the first MP session; (*iii*) Post-MP, at the end of phase B, 1–3 days after the last MP intervention); and (*iv*) follow-up (FU), at the end of phase A_2_, 1 month after the last MP session (Fig. 1).

### MP Interventions

The stages of the intervention related to the preparation of the MP tasks were based on the study of Santos-Couto-Paz [30]. These stages feature a continuous effort intensity, considering the principle of training intensity and specificity, and the cognitive and associative stages of motor learning. In this intervention model, the self-perception of the kinesthetic sense (first-person perspective) and the verbal description of each movement after the MP task were used to highlight learning. Hence, the volunteer uses cognitive and associative strategies (attention to processing the proprioceptive cues of the intended action) to extensively repeat the MP tasks, not focusing on one single movement, but rather on the task as a whole, making the task automatic to perform the movement [28, 30, 40].

In Group A, the MP was performed from a first-person perspective, wherein the difficulty of the motor task was increased during the sessions according to the continuum of intensity reported by the volunteers (Table 1). During the MP sessions, the volunteers from Group A imagined each task 10 times and, subsequently, were instructed to describe the movements of each joint (hip extension, knee extension, knee flexion, plantar flexion, etc.) imagined during the oriented-gait functional task. In contrast, volunteers from Group B imagined a number of non-motor tasks (Table 1) a total of 10 times each, and were subsequently instructed to describe each task. All MP sessions were guided by Researcher I (physical therapist).

**Table 1:** The first-person perspective mental practice tasks applied during the intervention-phase sessions

Group A	Group B
Sitting down and arising from a chair	Imagining and thinking of life goals
Walking with the prosthesis	
Walking fast	Imagining trips
Jumping over obstacles	
Running	
Walking up a staircase	Imagining and thinking about one’s family relationships
Walking up a ramp	
Walking and running in a “zig-zag” manner	
Walking down a staircase	Thinking and remembering moments of happiness
Walking down a ramp	

Group A: Oriented-gait motor functional tasks according to a continuum of intensity and effort; Group B: non-motor tasks

After each MP session, all volunteers were instructed to report their subjective perception of the effort related to the MP according to the Borg Scale [30]. In addition, Group A, but not Group B, performed gait training after the MP session in order to emphasize the movement tasks imagined. Each MP session had a duration of 40 min, and was performed 3 times per week over 4 weeks. Thus, a total of 12 sessions were conducted in a quiet and controlled environment by using objects and obstacles that replicated the gait variation. All volunteers imagined the same tasks in the same order as described in Table 1. Although they are the same for all tasks, both the MP and gait training were performed according to the individual efforts and limitations of walking.

### Gait Evaluation

The gait performance related to each stage (BL, Pre-MP, Post-MP, and FU) was evaluated using a force platform (AMTI, OR6-7; Germany) embedded into the lab floor. Each volunteer was instructed to walk along a 10 m linear trajectory, in his own footwear, at his natural cadence. A valid step was considered a step in which the prosthetic foot is directly hitting the force platform, with the entire foot making contact on the platform (not touching the platform edges). In this study, only the prosthetic limb was evaluated, since the MP protocol focused on the movement related to this limb. The data were low-pass Butterworth filtered (null phase), with a cut-off frequency of 50 Hz. The average values over 5 valid repetitions of walking were analyzed by a physical therapist (Researcher II), who had not participated in the MP sessions. The software DasyLab® version 10.0 (Dasytec, USA) was used to plot the force curves vs. time.

The first peak of the vertical GRF variable (V1 peak) was selected as the primary outcome measure due to its importance in the evaluation of gait performance, determined from the manner in which the individual touches the ground (initial contact: heel strike) [6, 37, 38]. As secondary outcome measures, the following variables from the GRF analysis of the prosthetic limb were included: the second peak of the vertical force (V2: propulsion phase), the first (AP1: related to braking capacity) and second anterior-posterior peaks (AP2: acceleration), the medio-lateral force (ML; prosthetic foot position), and finally, the time duration of the support phase [1, 2, 38, 39].

To determine the characteristics of the data, an independent researcher (III), not involved in the group assignments, established a database and performed statistical analysis using SPSS for Windows software (version 13.0; SPSS Inc., Chicago, IL). The Kolmogorov Smirnov test, with alpha = 0.05, indicated non-Gaussian distributions for the different kinetic variables. Thus, non-parametric tests with alpha = 0.05 were used to perform inter- and intra-group comparisons for the null hypothesis of no difference between the populations. Initially, each GRF variable (V1, V2, AP1, AP2, ML, and the time duration of support phase) in Groups A and B was compared by using the Mann-Whitney test for the BL and Pre-MP evaluations. For intra-group comparisons, the Friedman test was used to compare each variable among the 4 phases (BL, Pre-MP, Post-MP, and FU). If the *p*-value was significant (*p* < 0.05), the Wilcoxon test was used, along with post-hoc Bonferroni correction, to identify the differences across evaluations.

## Results

The Friedman test was applied to compare the gait evaluation in each group and revealed no statistical differences in all GRF variables among the BL, Pre-MP, Post-MP and FU stages in Group B (V1: *p* = 0.472; V2: *p* = 0.32; AP1: *p* = 0.90; AP2: *p* = 0. 27; ML: *p* = 0.90). Further, no significant difference was observed in the time duration to the support phase (Table 2, *p* = 0.90), indicating no gait improvement in Group B during the study. In addition, no significant difference was observed between Group A and Group B at the beginning of the BL stage (Table 2, Mann-Whitney test: *p* > 0.2). In both groups, the V1 peak of the vertical GRF was close to 90% of the body weight, while the magnitude of the V2 peak was close to 60% (Table 2). Hence, the gait evaluation indicated homogeneity of the groups. Moreover, the Wilcoxon test results indicated no difference between the BL and pre-MP phases in Group A (*p* > 0.92). Therefore, no gait improvement was observed before the first MP intervention.

**Table 2:** Gait evaluation results

	Phase	Ground Reaction Force (% of body weight)					Duration of Support (s)
		V1	V2	AP1	AP2	ML	
Group A	Baseline^**†**^	93.8 ± 5.1	63.3 ± 3.3	-13.6 ± 0.7	10.2 ± 0.4	3.3 ± 0.3	0.420 ± 0.074
(*n* = 10)	Pre-MP *	89.6 ± 7.2	66.4 ± 7.3	-19.4 ± 0.5	10.5 ± 0.7	3.1 ± 0.4	0.434 ± 0.088
	Post-MP * ^**‡**^	77.2 ± 6.2	79.4 ± 3.0	-11.9 ± 3.4	12.8 ± 2.2	2.6 ± 0.2	0.640 ± 0.054
	Follow-up^**‡**^	64.9 ± 3.2	74.0 ± 2.8	-13.2 ± 0.6	12.8 ± 0.6	2.2 ± 0.1	0.647 ± 0.090
Group B	Baseline^**†**●^	90.9 ± 3.7	62.5 ± 1.5	-13.2 ± 0.4	9.9 ± 0.4	3.1 ± 0.2	0.421 ± 0.029
(*n* = 5)	Pre-MP^●^	89.9 ± 6.9	62.4 ± 2.3	-22.8 ± 2.9	10.2 ± 0.5	3.2 ± 0.3	0.431 ± 0.094
	Post-MP^●^	89.9 ± 4.2	67.2 ± 3.3	-24.4 ± 4.7	9.2 ± 1.2	3.3 ± 0.2	0.431 ± 0.016
	Follow-up^●^	86.5 ± 3.9	62.3 ± 2.0	-23.4 ± 2.5	9.9 ± 0.4	3.4 ± 0.2	0.442 ± 0.095

The data are presented as mean values (± standard deviation) of the first and second peak of the vertical (V) and anterior-posterior (AP), and medio-lateral (ML) ground reaction force (GRF) variables and the time duration of the support phase over the prosthetic limb for Group A and Group B during gait evaluation

*MP* mental practice

†Mann-Whitney test (*p* > 0.20)

*Wilcoxon test (*p* = 0.002)

‡Wilcoxon test (*p* > 0.80)

●Wilcoxon Test (*p* > 0.89)

**Fig. 2:**
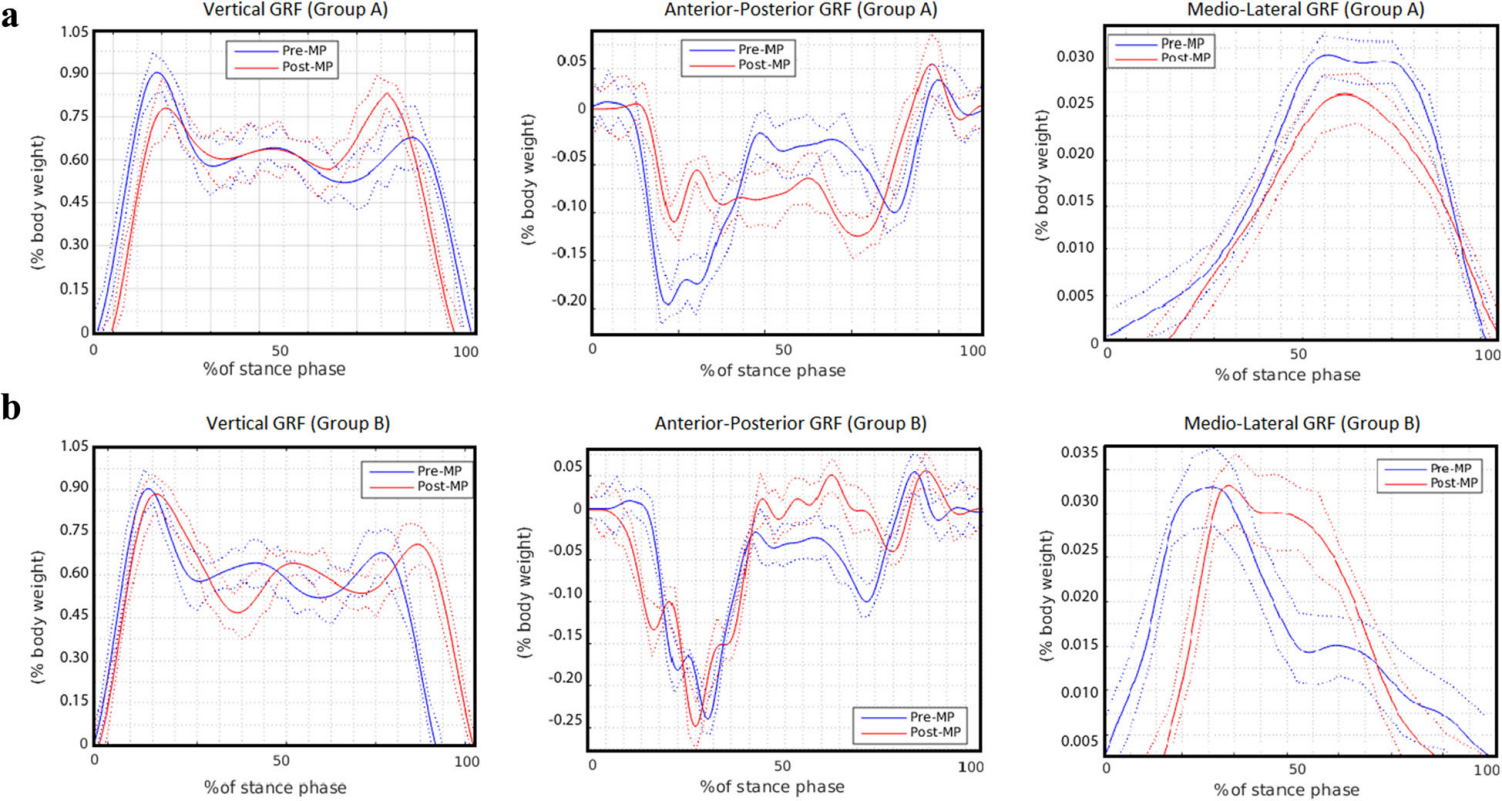
Gait evaluation over the 5 valid repetitions of the walking test. The mean curves (± standard deviation) of the vertical, anterior-posterior, and medio-lateral ground reaction forces (GRF) variables obtained for the prosthetic limb acquired just before mental practice (MP) intervention (Pre-MP: *blue*) and after MP intervention (Post-MP: *red*). **a** Group A; **b** Group B

Figure 2 shows the average GRF curves for Groups A and B obtained just before the MP (Pre-MP: blue line) and after MP intervention (Post-MP: red line). In Group A (Fig. 2a), the V1 peak of the vertical force during the Pre-MP phase indicated a magnitude close to the body weight (89.6%, Table 2). After the intervention, the V1 peak decreased to 77.2%, which was significantly different from that observed in the Pre-MP phase (Wilcoxon test, *p* = 0.002). On the other hand, the V2 peak increased from 66.4% to 79.4% after the MP intervention, enhancing the vertical force to promote limb advancement (*p* = 0.002). At the final FU stage, 1 month after the intervention, the vertical GRF values of did not differ compared to those obtained in the Pre-MP phase (Table 2, *p* > 0.80). In contrast, for Group B (non-oriented gait task, Fig. 2b), no difference was observed between the respective vertical GRFs obtained during the Pre-MP and Post-MP intervention phases, or after the FU stage (Table 2, *p* > 0.9).

In Group A, the negative first peak of the anterior-posterior GRF during the Post-MP intervention (AP1: red line in Fig. 2a) was a magnitude (11.9% of the body weight) lower than that observed at the beginning of the support phase during the Pre-MP stage (19.4%), with significant differences in the AP1 peak values (Wilcoxon test, *p* = 0.002). Moreover, the AP2 peak was increased at the end of the support phase, (*p* = 0.03). Furthermore, the magnitude of the medio-lateral force during the Post-MP stage (25.8%) was lower than that obtained during the Pre-MP intervention (31.3%) and was still decreasing after the FU stage (21.5%). On the other hand, Group B showed similar average Pre-MP and Post-MP curves for both the anterior-posterior and medio-lateral GRFs (Table 2, Wilcoxon test, *p* = 0.98).

With regard to the time duration of the support phase (Table 2), in Group A, a significant increase was observed, from 0.434 s (Pre-MP) to 0.640 s (Post-MP; *p* = 0.002). This result was maintained at 1 month after the intervention (FU; 0.647 s). However, no such enhancement occurred in Group B, with no significant changes observed among the 4 stages (*p* = 0.896).

## Discussion

In this study, MP, based on motor imagery tasks, was applied as a rehabilitation technique to improve the functional movements of gait in transtibial amputees. We applied the MP in the first-person perspective to enhance kinesthetic information and motor planning by focusing on the task as a whole, as suggested by Malouin et al. [28], Santos-Couto-Paz et al. [30], Stinear et al. [32], and Boutin et al. [40]. The gait-oriented MP was performed by considering the effort, intensity, and specificity of the tasks, as well as the motor learning stages. Moreover, the MP intervention used in our study focused on the benefits of clinical rehabilitation on locomotors skills of lower limb amputees. This model of intervention is similar to that applied in the rehabilitation of motor neural injury, stroke, training performance in sport activities, or upper limb prosthesis users, providing functional gain on subsequent physical training [20, 22, 25, 27, 30, 31].

All gait-oriented tasks used during the MP were selected according to the main complaint and difficulties of the volunteers in performing activities of daily living, such as, for example, maintaining body progression and stability while walking with the prosthesis over obstacles and walking up or down a ramp or staircase. On the other hand, body progression during these gait activities are dependent of the amount of the GRF generated by the foot position at the initial contact, as reported by Winter [1], Perry and Burnfield [2], Eils et al. [6], Takahashi et al. [37], Verdini et al. [38], and Sanderson and Martin. [39]. Hence, the MP intervention was applied combined with gait practice to emphasize the foot position during the initial contact of stance, weight acceptance, and push-off phase to preserve body progression and stability to the next step. This approach provided potential improvement and increased self-confidence to control the prosthetic limb during the motor learning stages in gait rehabilitation. These findings are similar to that observed by Santos-Couto-Paz et al. [30] in upper limb rehabilitation after stroke.

The GRF parameters assessed in gait evaluation during the BL stage of the present study indicated that all volunteers exhibited a homogenous gait pattern prior to the intervention. Moreover, there was no gait improvement during the Pre-MP stage. In both Group A (gait-oriented MP tasks) and Group B (non-motor MP tasks), the V1 peak of the vertical force curves showed a magnitude closer to the body weight, which suggested less impact absorption. Furthermore, the results indicated that the GRFs of both groups showed asymmetric magnitudes between the weight acceptance and push-off phases. These findings are consistent with that observed in the studies by Winter [1], Eils et al. [6], Nolan et al. [15], Sanderson and Martin [39], and Zmitrewicz et al. [41], in which the GRFs in unilateral below-knee amputees were investigated during walking. However, only Group A showed significant changes after the gait-oriented MP intervention, with reduced asymmetry between the GRF magnitudes, especially between the V1 and V2 vertical peak forces. According to Takahashi et al. [37] and Sanderson and Martin [39], a reduction of asymmetry in the magnitude of the GRFs reflects improvement of the functional performance of gait in lower limb amputees. This indicates that the MP provides advances in the clinical aspect of gait rehabilitation when included in the training program.

The gait-oriented MP applied in Group A significantly decreased the V1 peak force and increased the V2 peak (*p* = 0.002), indicating better impact absorption and propulsion capacity, respectively. Such findings, in terms of the V1 and V2 peaks, were considered as gait improvement in various previous rehabilitation procedures [8, 10, 13, 37, 39, 41]. In addition, the vertical GRF during the FU stage remained very close to those observed during the Post-MP stage (p ≈ 1). Thus, it appears that the gait-oriented MP sessions enhanced the motor function as a result from the adaptation and appropriate positioning of the prosthetic foot on the ground during the stance and the push-off phases, as suggested by Nolan et al [15], Bakker et al. [33], and Sanderson and Martin [39].

Only in Group A, the first negative peak of the anterior-posterior GRF (AP1) showed a significant decrease (*p* = 0.002) between the Pre-MP and Post-MP stages, whereas the second peak (AP2) increased (*p* = 0.002). This gait pattern persisted until the FU stage. These results indicate that the MP intervention provided the ability of the amputee to effectively adjust the propulsive force on the prosthetic side to improve body progression during push-off phase. Moreover, according to Vanicek et al. [5], Sanderson and Martin [39], and Zmitrewicz et al. [41], minimizing the asymmetry between the braking (AP1) and propulsion (AP2) capacities generated by the prosthetic limb leads to improve cadence. On this base, our findings suggest that the use of mental organization and motor planning to control the prosthetic limb helps to maintain body progression to the next step, as pointed out by Bakker et al. [33].

The medio-lateral GRF was significantly reduced in Group A (*p* = 0.002) when comparing the value between the Pre-MP and Post-MP stages. Such data represent the appropriate prosthetic foot position on the ground as well as stub-socket-heel-foot coupling during the single limb support phase, as observed by Schmalz et al. [13], Mattes et al. [14], and Zmitrewicz et al. [41]. Our findings also suggest better balance control and proprioception of the prosthetic limb on the ground to support body weight. According to previous studies [8, 10, 12, 41–43], better control of the prosthetic limb can help avoid erroneous positions of the foot (such as adduction or abduction), and can consequently increase the stability and time duration of the support phase, as observed in our study.

In the present study, a significant increase (*p* = 0.002) in the time duration of the support phase of the prosthetic limb in Group A was observed between the pre-MP (0.420 s) and post-MP (0.640 s) stages. This was not observed in Group B. In line with the findings reported by Kuo et al. [3], Baker and Hewison [8], Breakey [12], Mattes et al. [14], Nolan et al. [15], and Zmitrewicz et al. [41], the reduced time duration observed during the prosthetic limb support evaluation before MP may be associated with the instability or loss of proprioception to discharge body weight on the prosthetic side. On the other hand, rehabilitation procedures during gait training can enhance the proprioception and stability, and increase the time duration of the support phase up to 0.700 s, as pointed out by Kuo et al. [3] Baker and Hewison [8], and Schmalz et al. [13]. Thus, our results concerning gait-oriented MP to improve gait performance are consistent with those studies [3, 8, 12–15, 41].

This study was design to apply gait-oriented MP tasks combined with gait training to improve locomotor skills during gait. The MP tasks were based on gait activities and the intervention was performed for 40 min, 3 times per week, over 4 weeks. This approach resulted in a significant improvement of the functional performance of gait by reducing the asymmetry of the GRF variables. According to Jackson et al. [24], performance of MP for 5 days resulted in a gain equivalent to that of physical practice alone for 3 days. Indeed, the use of an MP strategy increases the efficiency of subsequent physical training [27, 44]. This suggests that the effects of MP are better when physical practice is added. Moreover, the gait-oriented training used in our study was consistent with the motor task-specific training procedure proposed by Hubbard et al. [45]. In this procedure, the motor-specific training intervention was shown to be effective for limb rehabilitation and everyday activity recovery. Therefore, from a clinical aspect, the MP intervention proposed in our study takes into account the fact that the amputees were familiar with the tasks and would be able to continue the treatment unassisted. Our results indicate that MP represents a complementary intervention for the rehabilitation of lower limb amputees and could be useful in clinical practice to enhance and control gait functions.

The motor-task MP combined with gait training proposed in the present study in lower limb amputees allowed the reestablishment of daily life functions and independent locomotion. Since the effect of mental training in Group A was maintained after 1 month (FU stage), the improvement in motor function may be related to the motor learning stages, considering the specificity of the MP tasks. However, the intact limb was not evaluated to assess the double support phase or to assess the aspects of symmetry during the load response and step length, and this represents a limitation of this study. Hypothetically, the imagination of motivating motor tasks as a whole can promote activation of bilateral cortical areas associated with the motor planning of the movement [17, 29, 46, 47]. Therefore, in future studies, the influence of motor training-based on MP should be explored in greater detail by using electroencephalography and functional magnetic resonance imaging. With these techniques, it is possible to evaluate the associative cortical or subcortical activation patterns during MP with gait training movements in lower limb amputees.

## Conclusion

The use of MP combined with gait training allowed the transtibial amputees in our study to reestablish independent locomotion. MP intervention based on gait-oriented functional tasks generated a significant improvement in gait performance, reduced the asymmetry in the magnitude of the GRFs, and enhanced the impact absorption, propulsion capacity, and balance control of the prosthetic limb during support. Since the effects of MP were preserved after 1 month of follow-up, the improvement can be speculated to be related to the specificity of the gait-oriented tasks. In turn, this indicates that MP provides advances in the clinical aspect of gait rehabilitation when included in the training program. Therefore, functional-oriented MP as a complementary intervention for the rehabilitation of lower limb amputees could be useful in clinical practice to enhance and control gait functions.

## Abbreviations

AP1: First peak of the anterior-posterior force
AP2: Second peak of the anterior-posterior force
BL: Baseline stage
CNS: Central nervous system
FU: Follow-up
GRF: Ground reaction force
MIQ-RS: Motor Imagery Questionnaire-Revised, Second Edition
ML: Medio-lateral
MP: Mental practice
Post-MP: After mental practice
Pre-MP: Before mental practice
V1: First peak of the vertical force
V2: Second peak of the vertical force
